# Structural Magnetic Resonance Imaging Brain Age Investigation in Athletes with Persistent Postconcussion Syndrome

**DOI:** 10.1089/neur.2024.0094

**Published:** 2025-01-31

**Authors:** Samuel Guay, Camille Charlebois-Plante, Sophie-Andrée Vinet, Marie-Eve Bourassa, Louis De Beaumont

**Affiliations:** ^1^ University of Montreal, Montreal, Canada.; ^2^ Centre de Recherche, Hôpital du Sacré-Cœur de Montréal, Montreal, Canada.; ^3^ Université du Québec à Montréal, Montreal, Canada.

**Keywords:** brainAGE, MRI, postconcussion syndrome, SHAP, sports-related concussion, traumatic brain injury, XAI

## Abstract

Brain age prediction algorithms using structural magnetic resonance imaging (MRI) estimate the biological age of the brain by comparing it to a normal aging trajectory, allowing for the identification of deviations that may indicate slower or accelerated biological aging. Traumatic brain injury (TBI) and sports-related concussion (SRC) have been associated with greater brain age gap (BAG) compared to healthy controls. In this study, we aimed to investigate BAG in athletes suffering from persistent postconcussion syndrome (PCS+) compared to PCS− athletes, and used SHapley Additive exPlanations (SHAP), an explainable artificial intelligence framework, to provide further details on which specific features drive the brain age predictions. Brain age was derived from T1-weighted MRI images in a cohort of 50 athletes (24 with PCS+) from 22 to 73 years old from the general population. The results revealed that athletes with PCS+ had a brain age approximately 5 years older than the PCS− athletes, with no clinical variable associated with it. Exploratory analyses also showed a greater brain age in athletes who self-reported five or more SRCs. Regarding SHAP, the third ventricle was found to be the most informative feature in the PCS+ group, while the superior temporal sulcus posterior area was more informative in the PCS− group. This study demonstrated the potential of using brain age and explainable artificial intelligence frameworks to study athletes with PCS. Further research is needed to explore the underlying mechanisms driving brain aging in this population and to identify potential biomarkers for early detection and intervention.

## Introduction

In the majority of cases of mild traumatic brain injuries (mTBI) and sports-related concussions (SRC), patients make a complete clinical recovery. Unfortunately, for a minority of patients, persistent and disabling symptoms will become chronic, leading to more complex outcomes, such as persistent postconcussive syndrome (PPCS) or, even worse, chronic traumatic encephalopathy.^1–3^ PPCS is usually defined clinically as a chronic condition in which SRC symptoms related to physical, cognitive, emotional, or sleep domains persist for more than 3 months.^4,5^ mTBI, SRC, and more recently repetitive head impacts have all been associated with long-lasting structural and functional brain changes which tend to worsen with age.^6–8^ While this conclusion is often reached in asymptomatic concussed athletes, this also holds for patients with PPCS.^2,9^ Yet, neuroimaging studies have not identified consistent brain regions specific to head traumas at the group level, reflecting substantial within-group heterogeneity.^10^ More importantly, repetitive head impacts and TBI increase risks for neurodegenerative disease and accelerated aging of the brain.^10–13^

A recent, popular data-driven approach to studying the so-called accelerated aging of the brain in various diseases is the brain age gap (BAG) estimation method.^14–16^ Brain age refers to the concept of estimating the biological age of the brain based on neuroimaging data, such as magnetic resonance imaging (MRI) scans, and comparing it to the individual’s chronological age. Several machine learning approaches have been used to accurately predict brain age, and the discrepancy between the predicted age and the chronological age allowing to identify deviation from the typical aging trajectory observed in healthy controls, making it an age-adjusted biomarker of brain integrity.^14,15,17^ Studies investigating BAG in moderate-to-severe TBI,^18^ complicated mTBI,^13^ and mTBI^19–21^ have reported a positive BAG (i.e., indicating biologically older brains) in TBI patients, suggesting an increased burden that worsens with TBI and symptoms severity. Mayer et al.^22^ also reported an increased BAG in young contact sport athletes. While studies have explored BAG in a wide range of TBI severity, there is still limited understanding of the BAG in PPCS athletes.

While solely focusing on the outcome of brainage models (i.e., the BAG) has proven to be useful as an individualized biomarker,^15^ leveraging an explainable artificial intelligence framework offers additional benefits by providing transparency, clinical interpretability, and aid in hypothesis generation. SHapley Additive exPlanations (SHAP) is one of these frameworks that can help unravel complex machine learning algorithms such as XGBoost models that predict age based on MRI.^14,23^ This method computes SHAP values for all features for each individual, revealing the direction and magnitude of each feature’s impact at the individual and global levels.^24^ It enables clinicians to understand how the model arrived at its predictions and determine if it aligns with clinical knowledge, thus improving trust in algorithms that can have implications for patients such as mortality risks in intensive care units and cancer.^25,26^ In the context of brainage, SHAP enables to identify and compare the most informative brain features for age prediction at the group level (global feature importance) in addition to allow characterization of the relationship between SHAP values and the BAG (local feature importance).^27^ This method has the potential to offer a more comprehensive understanding of the connection between brain regions and the larger BAG observed in TBI patients.^13,18–22,28^

Our objectives with this study were two-fold: (1) To investigate the BAG in PPCS athletes of all ages recruited from the general population and (2) to explore how SHAP can provide fine-grained insights into the most relevant brain features to predict age using both local and global explanations of model predictions in the latter clinical population. Based on previous TBI studies,^13,18,19,21,28^ we hypothesized that former athletes suffering from prolonged PCS would exhibit greater BAG in comparison to athletes with a history of SRC without PCS.

## Material and Methods

### Participants

All individuals involved in this study were former athletes recruited through University Athletic Alumni associations and Montreal medical services involved in the management of SRC. Participants were included if they met all of the following criteria: a history of repeated head impacts and concussion having occurred at least 1 year prior, no present alcohol and/or substance abuse behavior; no medical condition requiring daily medications or radiotherapy (malignant cancers, diabetes, and/or other cardiovascular diseases); and no contraindications to MRI. Based on a comprehensive clinical interview performed by a certified neuropsychologist and the validation DSM-IV-TR criteria for PPCS, also in line with the ICD-10 criteria for postconcussion syndrome,^29^ study participants were divided into two distinct groups: those manifesting PPCS (postconcussion syndrome [PCS+]) and those without PCS (PCS−). Data acquisition procedures were disrupted due to the COVID-19 pandemic, but a final sample of 50 participants with neuroimaging data was gathered, with 24 individuals in the PCS+ group and 26 in the PCS− group, which included 8 individuals from another project that matched the criteria for the PCS− group. The study was approved by the CIUSSS du Nord-de-l’Île-de-Montréal Research Ethics Committee, and all participants provided written informed consent and were financially compensated for their participation.

### Neuroimaging data acquisition and preprocessing

Structural T1-weighted images were acquired using a Siemens PRISMA or Trio 3.0T scanner (Siemens Healthcare, Erlangen, Germany) with a 3D magnetization prepared rapid gradient echo (MPRAGE) sequence using the following parameters: TR = 2300 ms, TE = 3.2 ms, FA = 8°, TI = 900 ms, and voxel size = 1 mm isotropic. Raw images were visually checked for artifacts at the scanner console, and participants were rescanned as necessary. DICOM files were converted into NIFTI files, named and reorganized following the brain imaging data structure (BIDS) specification v1.8.0 using dcm2bids.^30–32^ The FreeSurfer^33^ recon-all BIDS app^34^ with v7.4.1 was used in conjunction with Apptainer^35,36^ on an HPC cluster to ensure reproducibility and to automate the workflow. After having performed the recon-all command, we extracted the cortical thickness, area, and volume using the Human Connectome Project multimodal parcellation (HCP-MMP1)^37^ using specific HCP-MMP1 annotation files.^38^ We used this fine-grained cortical atlas as the pretrained models were based on the 360 data points for each measure (180 regions of interest [ROIs] per hemisphere). All raw and preprocessed images were carefully inspected, reprocessed as needed, and outliers were assessed based on Euler number output by FreeSurfer that was shown to be a reliable quality metric.^39^ One outlier in the PCS+ group was found and removed. Finally, the same BIDS app was used to aggregate in TSV files for all individuals the relevant features to compute brain age, namely the 38 FreeSurfer aseg stats and the 1080 data points from the HCP-MMP1.

### Brain age prediction

As our sample size is limited and composed of six females, we opted for sex-specific pretrained machine learning models available online at https://github.com/tobias-kaufmann/brainage. These gradient tree boosting^40^ models have been proven useful in highlighting patterns of apparent aging of the brain in several disorders including ADHD, schizophrenia, and dementia.^14^ These models were trained (*n* = 35,474), and validated (*n* = 5788 with brain disorders, *n* = 4353 healthy controls) on male or female brains exclusively, resulting in a separate pretrained xgboost model for each sex. Please refer to Kaufmann et al. (2019)^14^ for the complete training and validation procedure. In short, we fed the pretrained models matrices as input, in which each row contained the data from a given individual with the 1118 structural brain imaging features produced by the freesurfer pipeline, including the classical features such as total brain volume. To mitigate the systematic age bias known to over- and underpredict age of younger and older individuals respectively, we applied a bias correction procedure to control for the influence of chronological age on predicted brain age.^18,41^

### Global and local explanations through SHAP

Similar to the approach used by Ballester and colleagues,^27^ we derived subject- and global-level explanations through the extraction and visualization of SHAP values using the xgboost^42^ and SHAPforxgboost^43^ R packages. SHAP values represent the contribution of each feature to the prediction for a specific individual based on a game theoretical approach.^23^ Local SHAP explanations show the contribution of each feature to a specific individual prediction, highlighting how the feature values influence that particular outcome. We also computed a global measure of feature importance by averaging the absolute SHAP value for each feature across all individuals as it enables determining the most influential brain features in the model’s predictions. Global SHAP explanations show how much each feature contributes on average to the model’s predictions across the entire dataset, helping us understand the overall importance of features. We then summarized global and local explanations using a beeswarm plot and brain plots across groups in addition to exploring relationships between subject-level SHAP values and clinically relevant variables.

### Statistical analysis

All statistical analysis were performed using R 4.3.3,^44^ inside a reproducible conda environment.^45^ Unless otherwise stated, all statistical analyses used a significance level of *p* < 0.05 and corrected for multiple comparisons using the Benjamini−Hochberg approach as needed. The rstatix,^46^ permuco,^47^ and robustbase^48^ packages were used to perform all statistical and permutation tests with 100 000 permutations using the Freedman−Lane method^49^ on relevant variables, including the BAG, group, demographic and clinical variables, and SHAP values. One key advantage of permutation testing is that it does not require any assumption about the data distribution, making it robust, especially in small sample sizes.^47^ Exploratory analyses performed to guide hypothesis generation for future studies are identified as such.

## Results

### Demographic and clinical characteristics

Demographic and clinical information are summarized in Table 1. A total of 50 participants were included in the study, 24 PCS+ athletes (22 males) with a mean age of 43 ± 12 (min: 25, max: 64) years, and aged-matched 26 PCS− athletes (22 males) with a mean age of 47 ± 16 years (min: 22, max: 73), *p* = 0.25. The PCS− group 15.2 ± 3.6 (min: 7, max: 23) were slightly more educated than the PCS+ group 13.3 ± 2.7 years (min: 8, max: 18), *p* = 0.036. The PCS+ group self-reported more severe symptoms compared to the PCS− group on the MoCA, BDI, and BAI measures (*p* ≤ 0.001). Euler number was not statistically significant, indicating similar scan quality between both groups, *p* = 0.34.

**Table 1.** Demographic and Clinical Characteristics

Characteristic	Overall, *n* = 50^*a*^	PCS+, *n* = 24^*a*^	PCS−, *n* = 26^*a*^	*p* ^*b*^
Age (years)	45 ± 14	43 ± 12	47 ± 16	0.25
Education (years)	14.3 ± 3.3	13.3 ± 2.7	15.2 ± 3.6	0.036
Sex				0.67
Female	6 (12%)	2 (8.3%)	4 (15%)	
Male	44 (88%)	22 (92%)	22 (85%)	
Concussion number				0.14
≥5	17 (34%)	11 (46%)	6 (23%)	
1 − 4	33 (66%)	13 (54%)	20 (77%)	
MoCA	27.61 ± 2.75	26.22 ± 3.07	29.00 ± 1.41	< 0.001
BDI	10 ± 9	17 ± 8	3 ± 4	< 0.001
BAI	9 ± 9	13 ± 9	2 ± 4	< 0.001
Euler number	−17 ± 13	−15 ± 15	−18 ± 10	0.34

^a^ Mean ± SD; *n* (%).

^b^ Welch two-sample *t-*test; Fisher’s exact test.

BAI, Beck anxiety inventory; BDI, Beck depression inventory; MoCA, Montreal cognitive assessment; PCS+, with postconcussion syndrome; PCS−, without postconcussion syndrome.

### Predicted brain age and BAG

**FIG. 1.**
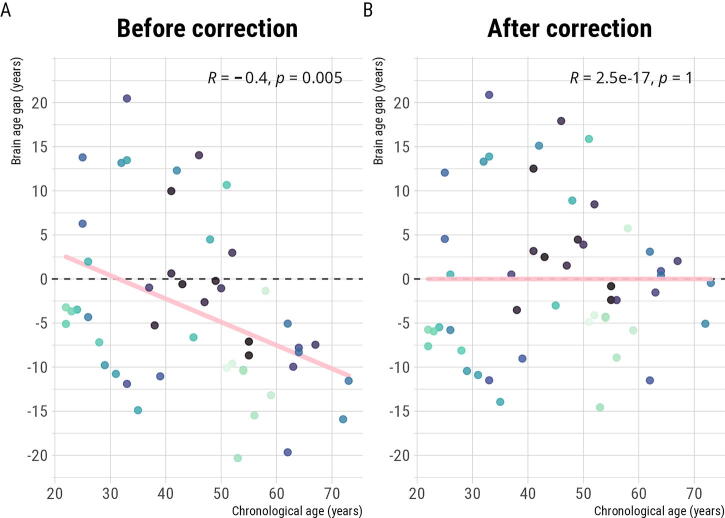
Effect of age bias correction on the brain age gap.

First, we addressed the systematic age bias occurring on the predicted age. The impact of this correction can be observed in Figure 1, where the correlation between the chronological age and the BAG (*[Corrected] Predicted age − Chronological age*) is mitigated.

**FIG. 2.**
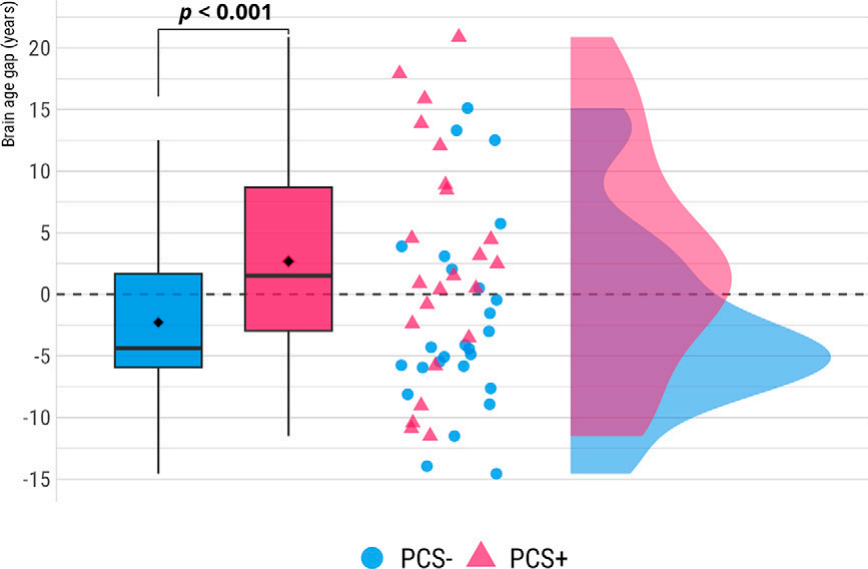
The brain age gap (BAG) is more pronounced in individuals with postconcussion syndrome (PCS+).

As expected, a greater BAG in the PCS+ group was observed compared to those in the PCS− group, β = 4.96, 95% CI [−Inf, 9.83], *t*(47) = 2.05, *p_r(>t)_* = 0.023, suggesting biologically older brains in the PCS+ group. PCS+ had a mean BAG of 2.68 years (SE = 1.77, 95% CI: [−0.87, 6.23]) compared to a mean BAG of −2.28 years (SE = 1.66, 95% CI: [−5.62, 1.06]) in the PCS− group. Figure 2 shows the difference and data distribution on raincloud plots.^50^

**FIG. 3.**
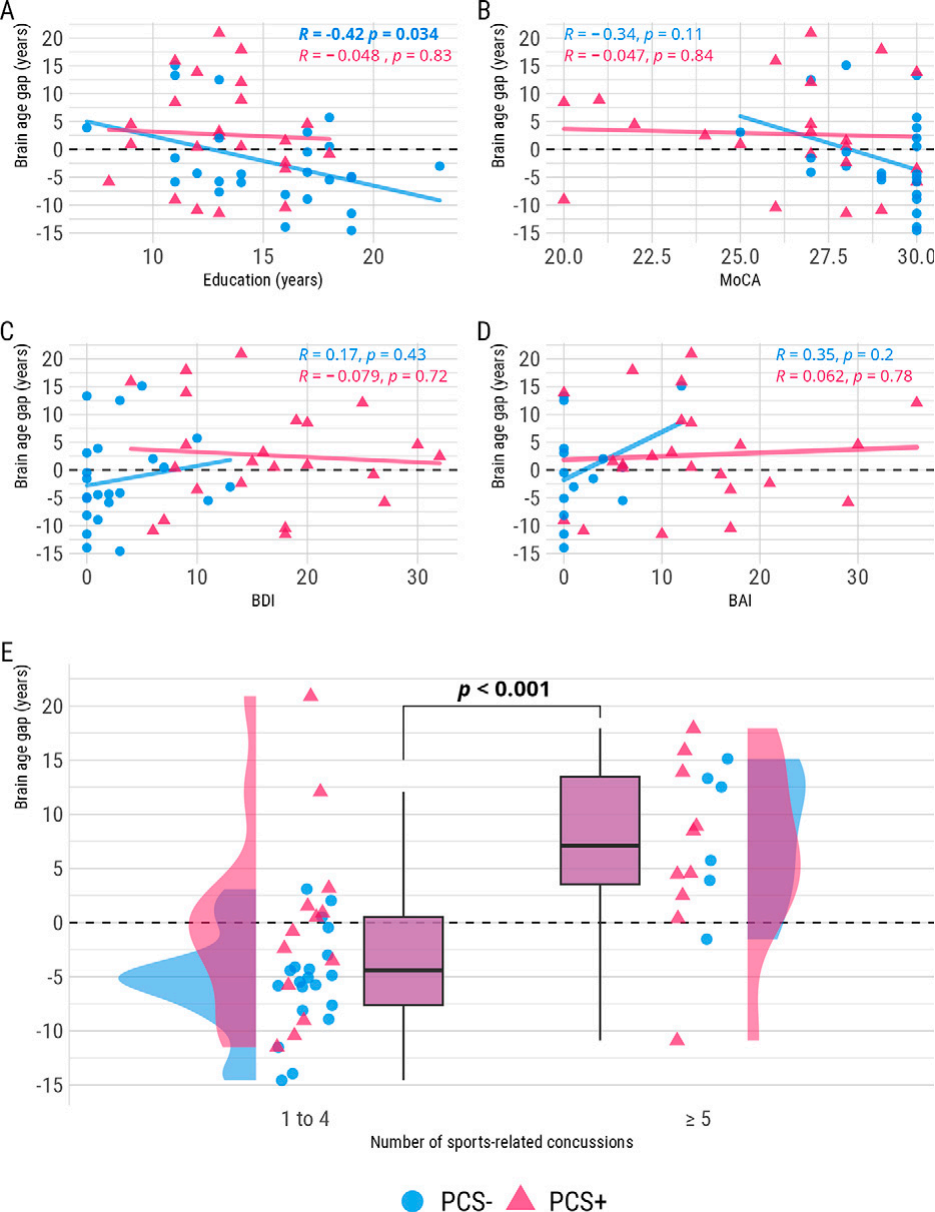
Univariate associations between potential predictors of brain aging and BAG. BAG, brain age gap; BAI, Beck anxiety inventory; BDI, Beck depression inventory; MoCA, Montreal cognitive assessment; PCS+, with postconcussion syndrome; PCS−, without postconcussion syndrome.

We then modeled associations between the BAG and sociodemographic separately for each group considering we did not have the power to test for interactions (Fig. 3). While MoCa, BDI, and BAI were not associated with the BAG (*p* > 0.05), a large, negative correlation was found between education and the BAG in the PCS− group only (*r* = −0.42, *t*[24] = −2.24, *p* = 0.034; Fig. 3A).

Regarding the number of SRCs reported, there was a statistically significant difference in the BAG, β = 10.61, 95% CI [6.18, 15.04], *t*(47) = 4.82, *p_r_* <.001, between individuals who reported ≥ 5 SRCs (−3.42 years, *SE* = 1.26, 95% CI: [−5.95, −0.88]) and those who reported 1 − 4 SRCs (7.19 years, SE = 1.81, 95% CI: [3.56, 10.83]). We could only test the main effect of SRCs regardless of groups number of participants with >= 5 SRCs in the PCS- group (6) but Figure 3E highlights the dispersion in both PCS+ and PCS− groups.

### Global and local explanations with SHAP

**FIG. 4.**
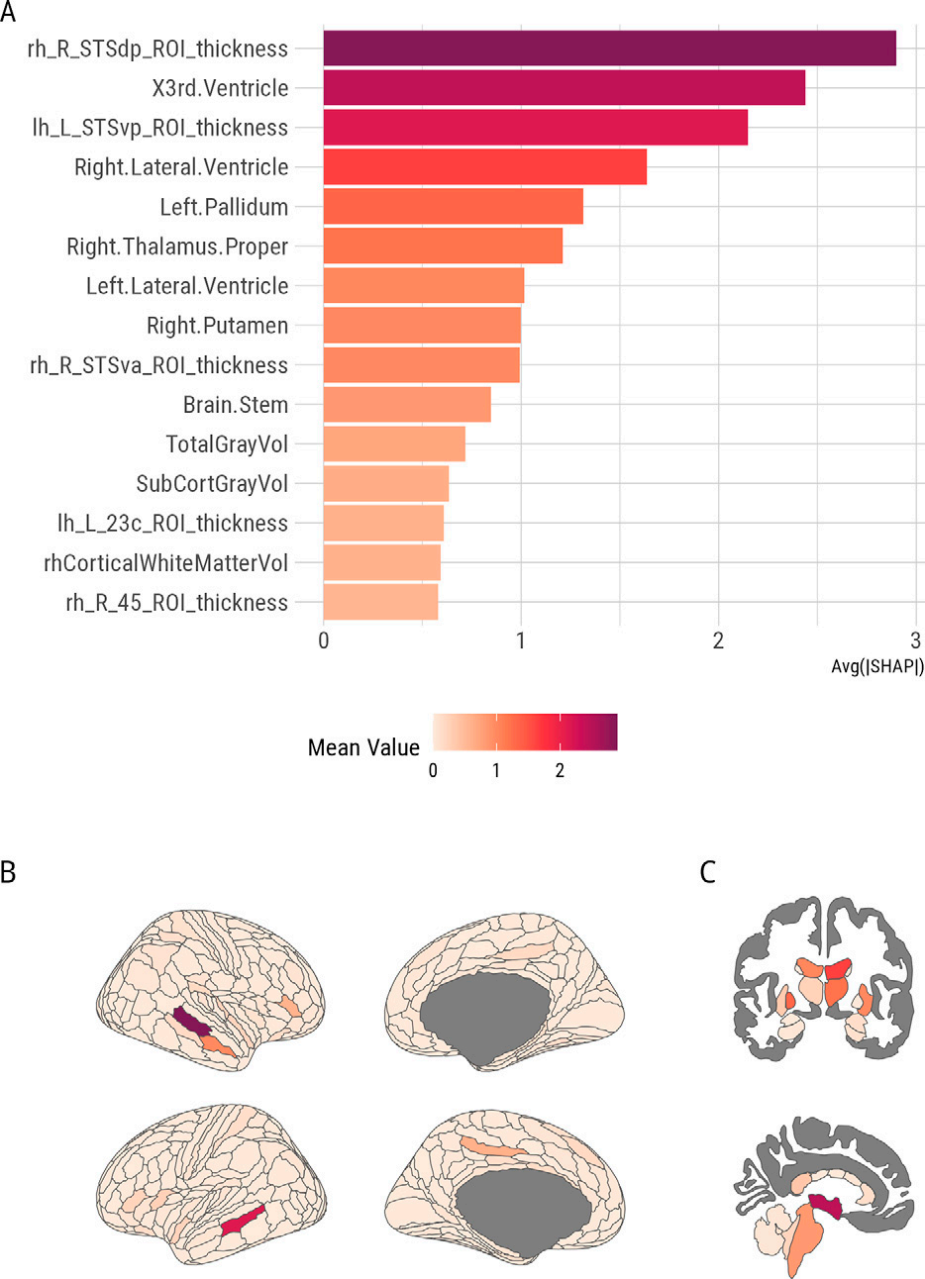
Most relevant features based on the global model structure. **(A)** The top 15 features in decreasing order of importance, while **(B)** and **(C)** show all the brain regions on HCP and aseg atlases respectively filled with absolute mean value. TotalGrayVol and SubCortGrayVol are not depicted in this figure. HCP, Human Connectome Project.

**FIG. 5.**
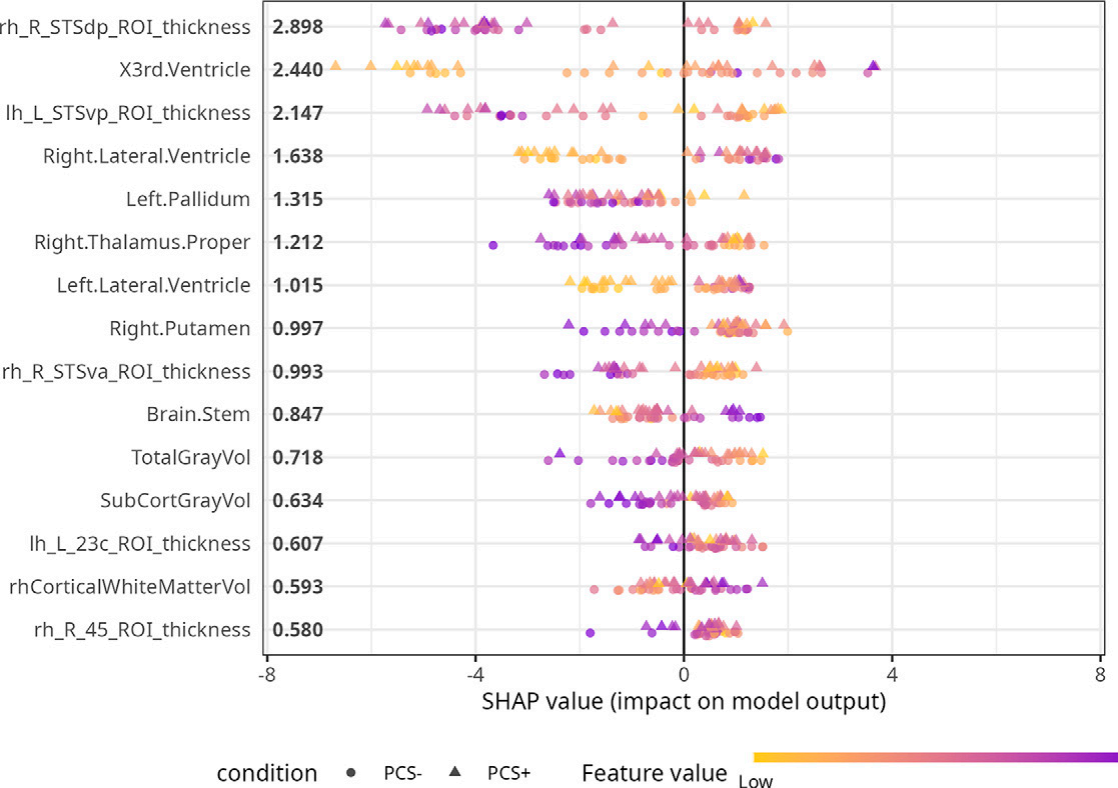
Order of the most relevant features in terms of their absolute mean value contribution in the PCS+ group **(A)** and PCS− group **(B)**. PCS+, with postconcussion syndrome; PCS−, without postconcussion syndrome.

To understand the spatial structure and contributions of all the features to brain age predictions we first summarized their global importance, which are depicted in Figure 4. While SHAP values of the top 15 unique, most informative features did not differ significantly between PCS+ and PCS− after applying FDR correction (all *p*-values > 0.05, Table 2), the order of the most relevant features in terms of their absolute contribution was different. Noteworthy, the third ventricle (volume) was the most informative feature in the PCS+ individuals (larger magnitude effect) compared to being third in the PCS− individuals, for whom the top spot was still the superior temporal sulcus (STS) posterior area (thickness) Figure 5.

**Table 2.** Group Comparison of SHAP Values Between PCS+ and PCS− Groups

Feature	Estimate^*a*^	Statistic^*a*^	*p* ^*a*^	*q* ^*b*^
rh_R_STSdp_ROI_thickness	−0.10	286.00	0.80	0.96
X3rd.Ventricle	0.44	337.00	0.46	0.94
lh_L_STSvp_ROI_thickness	−0.06	292.00	0.90	0.96
Right.Lateral.Ventricle	0.20	337.00	0.46	0.94
Left.Pallidum	−0.20	264.00	0.49	0.94
Right.Thalamus.Proper	−0.27	260.00	0.44	0.94
Left.Lateral.Ventricle	0.13	327.00	0.58	0.94
Right.Putamen	−0.15	247.00	0.30	0.94
rh_R_STSva_ROI_thickness	−0.02	296.00	0.96	0.96
Brain.Stem	0.11	335.00	0.48	0.94
*TotalGrayVol*	*−0.44*	*190.00*	*0.03*	*0.43*
SubCortGrayVol	−0.14	273.00	0.61	0.94
lh_L_23c_ROI_thickness	0.07	318.00	0.71	0.96
rhCorticalWhiteMatterVol	−0.12	274.00	0.63	0.94
rh_R_45_ROI_thickness	0.02	308.00	0.87	0.96

^a^ Wilcoxon rank sum test.

^b^ False discovery rate correction for multiple testing.

PCS+, with postconcussion syndrome; PCS−, without postconcussion syndrome; ROI, regions of interest.

Italic data reached significance threshold uncorrected for multiple comparisons (*p*-values).

**FIG. 6.**
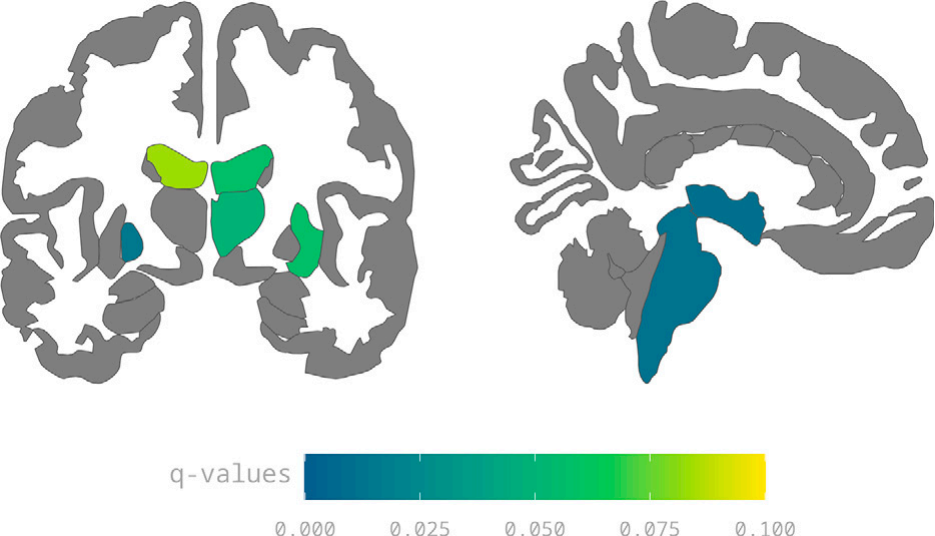
Global view and local view of the importance and effects of the features across all predictions.

To grasp how higher and lower values of each feature affected the predicted age in each individual based on their group, we first plotted a beeswarm plot. Figure 6 demonstrates that the pretrained models made predictions aligned with expectations in an aging brain. In general, higher predicted age corresponded to smaller values of STS thickness, total and subcortical gray matter volume, Putamen and Thalamus, and larger ventricles among others. Data distribution appears similar between the PCS+ and PCS− groups for all top 15 features. We then tested the interaction between the groups and SHAP values of the 15 features identified earlier in relation to the BAG to identify brain regions or features that had more significant contributions in one group compared to the other. Only the STS thickness in the right hemisphere × group interaction was significant before applying FDR correction, otherwise all *p*-values were > 0.05 (Table 3).

**Table 3.** Interactions Between 15 Most Relevant Brain Features × Groups (PCS+ and PCS−)

Feature	Estimate^*a*^	Statistic^*a*^	*p* ^*a*^	*q* ^*b*^
*rh_R_STSdp_ROI_thickness*	*2.17*	*2.06*	*0.04*	*0.67*
X3rd.Ventricle	0.06	0.09	0.93	0.98
lh_L_STSvp_ROI_thickness	0.80	0.74	0.47	0.98
Right.Lateral.Ventricle	0.30	0.25	0.80	0.98
Left.Pallidum	1.23	0.60	0.55	0.98
Right.Thalamus.Proper	1.53	0.79	0.43	0.98
Left.Lateral.Ventricle	1.59	0.98	0.33	0.98
Right.Putamen	−0.27	−0.11	0.91	0.98
rh_R_STSva_ROI_thickness	2.38	1.06	0.30	0.98
Brain.Stem	0.20	0.10	0.92	0.98
TotalGrayVol	0.18	0.03	0.97	0.98
SubCortGrayVol	−0.45	−0.15	0.88	0.98
lh_L_23c_ROI_thickness	−7.60	−1.03	0.31	0.98
rhCorticalWhiteMatterVol	0.14	0.03	0.98	0.98
rh_R_45_ROI_thickness	4.98	0.96	0.34	0.98

^a^ Robust linear regression with MM-type estimators.

^b^ False discovery rate correction for multiple testing.

PCS+, with postconcussion syndrome; PCS−, without postconcussion syndrome; ROI, regions of interest.

### Exploratory analyses

**FIG. 7.**
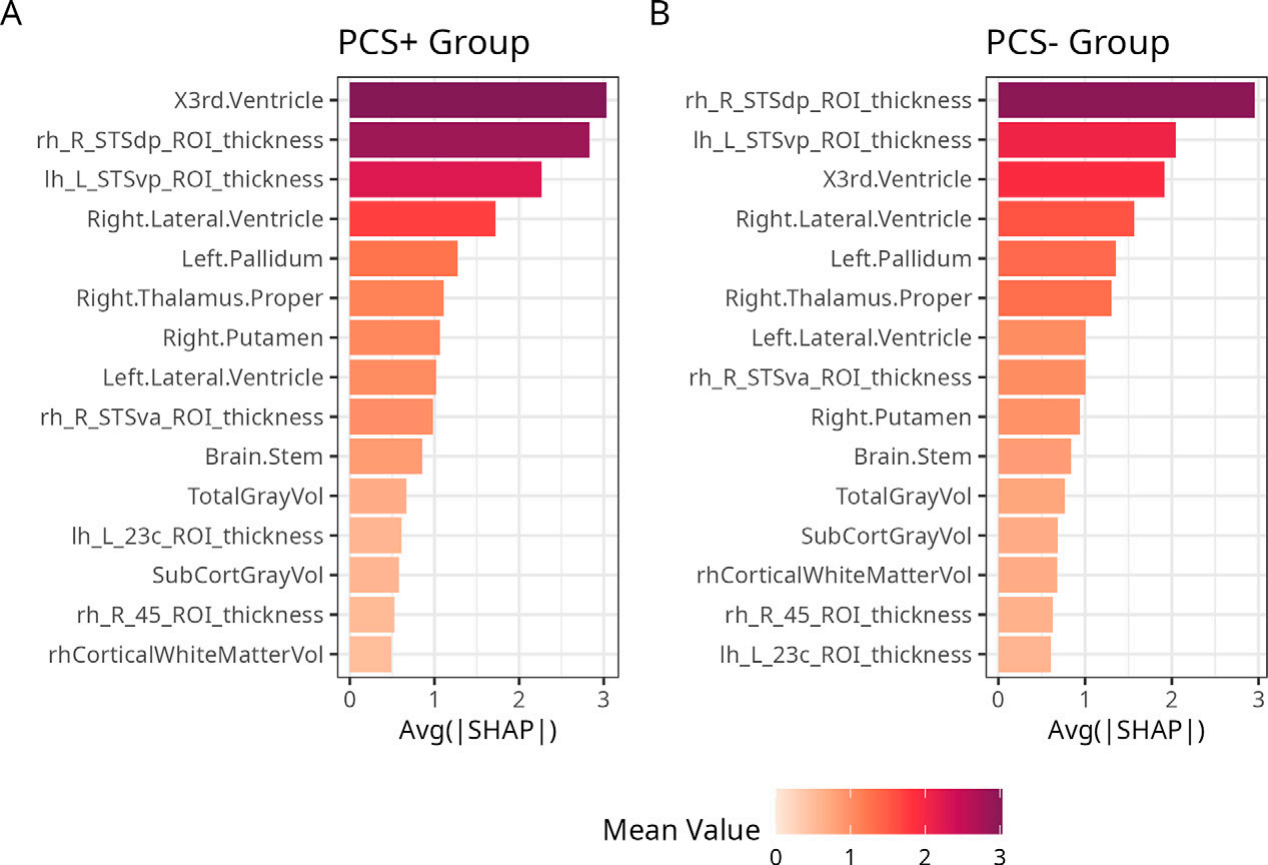
Brain features differences in mean SHAP values between 1 and 4 SRCs versus ≥ 5 SRCs. All regions showed have *q*-values > 0.10. TotalGrayVol and SubCortGrayVol are not depicted in this figure. SHAP, SHapley Additive exPlanations; SRC, sports-related concussion.

Considering the larger BAG found in athletes with at least 5 SRCs, we repeated the same SHAP group comparisons to explore potential differences regardless of PCS groups. Table 4 shows statistically significant differences in bold and Figure 7 highlights brain features with *q*-values > 0.10 to aid hypothesis generation for future studies.

**Table 4.** Group Comparison of SHAP Values Between 1 and 4 SRCs Versus ≥ 5 SRCs

Feature	Estimate^*a*^	Statistic^*a*^	*p* ^*a*^	*q* ^*b*^
rh_R_STSdp_ROI_thickness	−0.15	245.00	0.70	0.80
X3rd.Ventricle	**−2.27**	**123.00**	**0.00**	**0.01**
lh_L_STSvp_ROI_thickness	0.05	273.00	0.86	0.86
*Right.Lateral.Ventricle*	*−0.74*	*165.00*	*0.03*	*0.06*
Left.Pallidum	**−0.77**	**134.00**	**0.00**	**0.01**
*Right.Thalamus.Proper*	*−0.97*	*158.00*	*0.02*	*0.05*
Left.Lateral.Ventricle	−0.39	174.00	0.06	0.08
*Right.Putamen*	*−0.30*	*165.00*	*0.03*	*0.06*
rh_R_STSva_ROI_thickness	**−0.72**	**142.00**	**0.01**	**0.02**
Brain.Stem	**0.61**	**401.00**	**0.00**	**0.01**
TotalGrayVol	**−0.88**	**107.00**	**0.00**	**0.01**
SubCortGrayVol	**−0.71**	**112.00**	**0.00**	**0.01**
lh_L_23c_ROI_thickness	−0.13	229.00	0.47	0.58
rhCorticalWhiteMatterVol	0.28	318.00	0.26	0.35
rh_R_45_ROI_thickness	−0.02	249.00	0.76	0.81

^a^ Wilcoxon rank sum test.

^b^ False discovery rate correction for multiple testing.

SRC, sports-related concussion; SHAP, SHapley Additive exPlanations; ROI, regions of interest.

Bold data represent corrected for multiple comparisons (q-values).

## Discussion

Our main goal with this study was to investigate the presence of a greater BAG, a putative marker of overall brain health, among a cohort of concussed athletes with persistent PCS in comparison to concussed athletes without PCS. In our sample, PCS+ individuals’ brain age was estimated approximately 5 years older than the PCS− individuals’. This finding corroborates previous TBI studies that reported a greater BAG in TBI patients of varying severity.^13,18,19,21,22,28^ Additionally, our main findings are in agreement with previous findings of a greater BAG based on diffusion-tensor imaging metrics in TBI patients reporting at least three PCS complaints.^21^ It appears that brain age predicted from T1w or DTI is sensitive to PCS. Further investigation is needed to determine if a multimodal study can lead to improved prediction in concussed athletes and help identify individuals at higher risk for chronic complications. It is worth noting that the groups differed on some demographic and clinical variables which is expected considering the nature of the groups. The PCS+ reported more severe clinical symptoms, including anxiety and depression, lower MoCA scores, and level of education. These findings align with patients suffering from postconcussion syndrome. None of the clinical variables correlated with the BAG. However, education was negatively correlated with the BAG in the PCS− group only. This finding in controls only was previously reported in TBI^21^ as well as in a recent study on determinants of brain age in patients with HIV versus without that reported a negative correlation in controls without HIV only.^51^ Moreover, older-appearing brains were found in athletes reporting having sustained at least 5 SRCs relative to those with a history of 1 − 4 SRCs regardless of PCS group. This suggests that repeated SRCs are associated with a progressive increase in BAG. Conversely, a previous study on young athletes showed that the BAG was unrelated to either SRC history or years of exposure to contact sports.^22^ Apart from methodological variations, there was a substantial difference in the mean age of the recruited cohorts (20.1 ± 1.1 vs. 45 ± 14 years). Although future studies with larger samples are needed to validate our findings, one may raise the possibility of a synergistic effect of aging with a history of concussion on accelerated aging of the brain.

Our second goal was to explain the models behavior in a transparent way and gain insights from global- and local-level explanations with respect to the BAG. While many brainage models are available, we used proven sex-specific XGBoost models known to be more sensitive to clinical diagnoses.^14,52^ The global-level explanation revealed the same, most important brain features for brain age prediction that were found using the same method in a schizophrenia study regardless of diagnosis.^27^ Although there was a slight change in the order of importance, with the third ventricle being the most influential in the PCS+ group compared to the right hemisphere STSdp in the PCS− group, our analyses did not reveal any significant differences in SHAP values. This suggests that there was no change in the directionality or magnitude of the prediction between the PCS+ and PCS− groups, indicating similar prediction behavior in both groups. All regions being equally important in the prediction aligns with the fact that they are derived from the same population of individuals with SRCs. However, exploratory analyses uncovered a change in directionality between individuals with 1–4 SRCs and those with 5 or more SRCs. This implies that certain brain features, mostly subcortical structures and total gray matter volume, contributed to lower or higher predictions based on the number of concussions, rather than PCS symptoms. Interestingly, deep gray matter structures were found to be reduced in amateur boxers^53^ so it remains to be confirmed whether this is a contact-sports-specific finding. Moreover, longitudinal changes in ventricle size were also observed as young as in high school football athletes, suggesting that ongoing participation in collision-based sports may pose a risk where full recovery is not possible.^54^ This risk appears to manifest at multiple stages throughout an athlete’s career and warrants further detailed investigation.

Despite providing valuable insights into PCS+ athletes through data-driven approaches to data analysis, this study has several methodological limitations. First, caution should be taken when interpreting the results due to the small sample size and cross-sectional design, although robust methods such as permutation-based statistical tests were employed. Additionally, the absence of a control group consisting of nonconcussed former contact sports athletes hinders the ability to differentiate the effects attributed to an SRC history from those related to PCS. Furthermore, the etiology of PCS remains a subject of debate among experts.^55^ Given the overlapping nature of symptoms with other conditions, such as major depression, it is challenging to determine whether the symptoms arise from the physiological consequences of the injury or preexisting or postinjury physical and psychological factors. Future studies on the long-term consequences of PCS+ should consider including patient groups with similar symptomatology to better characterize the distinct effects of PCS+. Another limitation is the potential for recall bias in the self-reported number of concussions, which necessitated dichotomization due to the wide range of uncertainty. Although the decision to dichotomize the self-reported number of concussions may have reduced the statistical power of the study, it is crucial to note that the primary focus was not on the number of SRCs but on PCS+ athletes. Furthermore, those having more than five concussions tended to report numbers closer above 10. However, to maintain balance between the groups, a conservative limit of five concussions was used. The retrospective nature of the data introduces the potential for inaccuracies, which could affect the reliability of the study’s findings.

In conclusion, this study has demonstrated the potential of using MRI-derived brain age to study the effect of SRCs in athletes with an explainable, more transparent AI framework. Our approach differs from prevalent studies focusing on active elite or former professional athletes, as our inclusive methodology encompasses former athletes from the general population with a wider age range in order to capture the enduring effects of SRCs in individuals often underserved in specialized care.

## Abbreviations Used

BIDS: brain imaging data structure
mTBI: mild traumatic brain injuries
SRC: sports-related concussion
TBI: Traumatic brain injury
